# Strength exercise for balance and gait in HIV-associated distal symmetrical polyneuropathy: A randomised controlled trial

**DOI:** 10.4102/sajhivmed.v22i1.1268

**Published:** 2021-10-05

**Authors:** Abdulsalam M. Yakasai, Sonill Maharaj, Musa S. Danazumi

**Affiliations:** 1 Department of Physiotherapy, Faculty of Health Sciences, University of KwaZulu-Natal, Durban, South Africa; 2 Kano Zonal Office, Medical Rehabilitation Therapists Board, Kano, Nigeria; 3 Department of Physiotherapy, Faculty of Allied Health Sciences, Bayero University, Kano, Nigeria; 4 Department of Physiotherapy, Federal Medical Centre, Nguru, Yobe State, Nigeria

**Keywords:** HIV-associated neuropathy, balance, gait, strength training, outcomes, rehabilitation, antiretroviral therapy

## Abstract

**Background:**

HIV-associated peripheral neuropathy (PN) is a common neurological complication associated with HIV infection. Distal symmetrical polyneuropathy (DSPN) is the most commonly occurring type, which is associated with symptoms such as numbness, unsteady gait and, in some cases, muscle atrophy and weakness when myelinated nerve fibres are affected. If unmyelinated nerve fibres are affected, a painful neuropathy and autonomic symptoms may occur.

**Objectives:**

This research study assessed the effects of a strength exercise intervention on balance impairment and gait disturbance amongst individuals living with HIV-associated DSPN.

**Method:**

The study was a single-blinded, randomised controlled trial (RCT) with participants sourced from four HIV centres in Kano metropolis, Nigeria. The intervention was supervised and included progressive resistance exercise (PRE) (three 40-min sessions per week for 12 weeks) using a quadriceps bench (*n* = 44). The control group (CG) included the non-exercise group (*n* = 47). The two groups continued to receive routine care. Data were summarised and analysed using inferential statistics (SPSS version 20 program) with the alpha level set at < 0.05.

**Results:**

At 12 weeks, the results revealed significant improvement with regard to balance performance (*p* = 0.001) and walking ability (*p* = 0.001) in the training group. In contrast, no significant differences in balance (*P* = 0.677) or gait (*P* = 0.578) were observed in the CG.

**Conclusion:**

The findings suggest that PRE is beneficial for balance impairment and gait disturbance caused by neuropathy in persons living with HIV and receiving antiretroviral drugs.

## Introduction

Peripheral neuropathy (PN) is a neurological condition affecting the sensory, motor or autonomic peripheral nerves. The classification of PN is generally based on the cause or pathological features of the nerve fibres affected. It can be subdivided based on primary myelin or axonal damage into demyelinating or axonal PN.^1^ The global estimate of HIV-associated neuropathy varies widely from 1.73% to 69.4% in different HIV-infected populations.^2^ Sadly, PN was not only shown to be associated with HIV infection itself but in many cases also triggered by treatment with antiretroviral agents.^3^ Growing evidence of unwanted adverse effects of the early antiretrovirals (ARVs) led to the use of safer, later generation ART in many countries. Peripheral neuropathy nontheless still remains important as the most common neurological disorder of people living with HIV (PLHIV).^4^

A number of studies from resource-constrained settings including sub-Saharan Africa report that the risk of HIV-associated neuropathy amongst patients on ART to be high (between 30% and 64%).^5,6^ Indeed, HIV-associated distal symmetrical polyneuropathy (DSPN) is reportedly the most common neurological complication of HIV infection.^7^ Individuals living with DSPN experience pain, numbness, fatigue and muscle weakness.^8,9^ These symptoms may become debilitating with locomotor instability resulting in an altered gait, an increased rate and incidence of falls, and decreased quality of life (QOL).^10^ The falls are attributed to the balance and gait abnormalities.^11^

Gait requires balance, power and coordination of larger groups of muscles of the lower extremities to permit the body to be propelled forward in a rhythmic motion. This function is diminished or lost in persons with neuropathy.^12^

Walking is a complex task that requires the coordinated function of the musculoskeletal, neuromuscular and cardiopulmonary systems. As DSPN progresses, symptoms such as ‘numbness’, pins and needles and ‘pain’ in the soles of the feet worsen the disturbed gait.^13^ Functional limitations, psychological distress and the loss of independence further compound the disability of those with HIV-associated DSNP.^14,15^ These negative effects and the risk of injury impact the survival of affected individuals.^12,13^

Exercise is generally regarded as safe for HIV-infected persons because it does not compromise immune function, boosts functional capacity, strength, physical fitness, mood, well-being, and may ameliorate muscle wasting and lipodystrophy.^16,17^ Previous studies have investigated the effect of aerobic and resistance exercise individually or in combination on immune function, psychological factors, cardiorespiratory fitness, strength, body composition, QOL^17,18^ and the ART-induced metabolic complications of PLHIV.^19^ The findings from these studies reveal that moderate- to high-intensity aerobic exercise is safe and elicits favourable and beneficial changes in all the variables examined.

Physical therapy interventions have been shown to help address balance impairment, gait disturbance and to have a positive effect on the QOL of persons with complex chronic illnesses.^20^ In clinical practice, the physical therapists will use therapeutic resistance exercises that cause muscular contraction, microscopic damage/tearing of muscle, and repair that permits the restoration/increase of muscle power, tone, mass and endurance.^21^ A marked improvement in postural sway, joint flexibility, pain reduction, balance-stance, gait speed and QOL has been observed following ‘exergaming exercise’ and strength training by PLHIV who have postural and walking impairment.^17,22,23^ A recent study further suggests that regular strength exercise may reduce HIV-neuropathic pain.^24^

Studies of the role of exercise in the rehabilitation of those on ART and living with neuropathy are scarce.^24^ To the best of the authors’ knowledge, there is no data on the impact of progressive resistance exercise (PRE) in the management of balance impairment and gait disturbance in HIV-positive individuals who develop DSPN. This study investigated the effect of PRE on balance impairment and the walking ability of HIV-positive individuals on ART and living with neuropathy.

## Methods

### Research design

This was a single-blinded, pre-test, post-test randomised controlled trial (RCT).

#### Participants

Participants were patients living with HIV and attending local HIV centres. A neurologist performed a baseline physical assessment to confirm the presence of DSPN before enrolling the subjects in the study. The criteria for inclusion were: individuals aged 20–55 years, diagnosed with DSPN on ART from the ART clinics, able to ambulate unsupported and able to complete the 6-min walk test. The exclusion criteria were non-HIV related DSPN, serious cardiac pathology, musculoskeletal system pathology, loss of protective sensation of the feet, difficulty to walk without support, cerebrovascular accident and the following medical conditions such as diabetes mellites, high blood pressure (stage 2), hypoactive thyroid, vitamin B12 and folate deficiencies, co-infection with syphilis and chronic hepatitis C, the use of drugs known to cause PN, substance or alcohol abuse and kidney failure.

#### Population, site and sampling

The RCT included 102 participants on ART diagnosed with HIV-associated DSPN who attended four ART clinics situated in Murtala Mohammed Specialist Hospital (MMSH), Mohammed Abdullahi Wase Specialist Hospital (MAWSH), Infectious Diseases Hospital, and international clinics and hospitals using the purposive sampling technique. The choice of purposive sampling was to include HIV-infected individuals from various settings around Kano metropolis, with different languages and socio-economic backgrounds.

#### Sample size estimation

**FIGURE 1:**
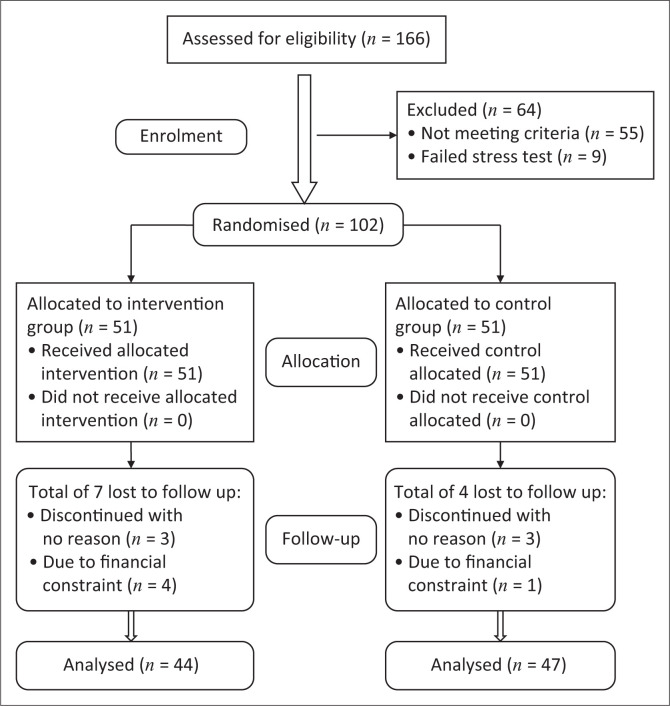
Recruitment and retention: Brief peripheral neuropathy screening tool.

The parameters to calculate sample size were set as follows: power of the study = 80%, alpha level < 0.05, groups = 2, effect size *f* = 0.32 (medium) and number of repeated measures = 3. A sample of 43 participants would be required in each group. The sample size of 86 was sufficient for this study; however the number of participants recruited was increased to 166 to accommodate the expected attrition. The participants were divided into two groups: PRE group and a control group (CG). An independent physiotherapist conducted randomisation using a computer random number allocation sequence. Based on the initial assessment, 55 participants were disqualified during the initial examination because of the absence of signs or symptoms of neuropathy and nine participants failed the stress tests, leaving 102 participants for randomisation into exercise and CGs. Ninety-one participants completed the study as shown in the consort flowchart of the study (Figure 1).

### Assessment and diagnosis

#### Subjective peripheral neuropathy scale

The Subjective Peripheral Neuropathy Scale (SPNS) was used to assess the presence of DSPN. Participants were asked to tick the box in front of the option that applied to them. The options were as follows: ‘always been normal’ to indicate the presence of the symptoms before but not present during the previous visit, or ‘currently absent’ if the symptoms had been there but were not present at the moment. The symptoms were graded based on the severity at that moment using the scales: 1–3, 4–6 and 7–10, which is converted into mild, moderate and severe, respectively. The symptoms assessed were as follows: muscular cramping, numbness in the feet or legs, tingling sensation, burning and aching sensations, and sharp stabbing or shooting pains. Symptoms of a sudden electric-shock-like sensation, especially during sleep, sensations in the feet whilst walking and the ability to differentiate cold from hot were also assessed.^25^

The Brief Peripheral Neuropathy Screening (BPNS) tool confirmed the presence of neuropathy, which is a validated and reliable tool used as both an objective and subjective method for the diagnosis of neuropathy, especially DSPN, amongst HIV-positive individuals.^26^ It can be administered by non-neurologists because of its simplicity, and it covers both signs and symptoms of neuropathy. The severity and the presence of the symptoms were scored using a scale of 1–10; 0 = no symptoms, 1–3 = mild symptoms, 4–6 = moderate symptoms and 7–10 = severe symptoms.

The following symptoms were assessed: burning, aching or pain; pins and needles; and numbness in feet or legs. The highest score was 6, with three points for each leg. The scores were converted into grades as follows: grade 1 = mild neuropathy, grade 2 = moderate neuropathy and grade 3 = severe neuropathy. A grade of ≥ 1 was provided when symptoms were present in both legs. The sense of vibration and the quality of the Achilles tendon reflex (TA) were the objective signs tested in the BPNS. The sense of vibration was evaluated using a 128-Hz tuning fork struck maximally and applied to the great toe at the distal interphalangeal joints bilaterally. Sense of vibration was scored as normal if the vibration was felt for > 10 s, mild loss if felt for 6 s–10 s, moderate loss if felt for < 5 s and severe loss if no vibration was felt. The reflexes of TA were assessed with the participant seated on a chair. The examiner then dorsiflexed the foot to 90° and struck the TA using a reflex hammer. The TA reflex was felt in the hand of the examiner as a contraction of the gastrocnemius muscle. The participant was then instructed to clench his or her fist, and the reflexes were graded as follows: 1 = hypoactive, 2 = normal, 3 = hyperactive, 4 = clonus and 0 = non-reactive.

### Study measures

#### Balance and gait assessment

The Tinetti Performance-Oriented Mobility Assessment (POMA) scale was used to evaluate the performance of the balance and gait of the subjects. It can be simply implemented using a hard armless chair. The POMA consists of two subscales: a balance subscale (POMA-B) and a gait subscale (POMA-G). The POMA-B evaluated the following items: 1 = sitting balance that is safe and steady, 0 = when lean or slide, 2 = rise, 3 = attempt to rise, 4 = immediate standing balance (first 5 s), 5 = ability to stand in one attempt, 6 = nudged (participant assumed a position with the feet as close as possible and the examiner pushed the participant slightly at the sternum with the palm of the hand three times), 7 = same position as item six but with both eyes closed, 8 = turning (participant turned 360°) and 9 = back to sitting position. Some items were scored as binary (0 = inability to perform, 1 = ability to perform), whilst others were scored as follows: 0 = inability, 1 = adaptive and 2 = normal. The POMA-B has a total of 16 marks; < 10 = poor balance ability, 10–13 = good balance ability and 14–16 = excellent balance ability.

The POMA-G evaluated the following items: 1 = initiation of gait (immediately after given the command, ‘GO’), 2 = same step length and height bilaterally, 3 = step symmetry, 4 = step continuity, 5 = path deviation, 6 = stability of the trunk and 7 = walking stance phase. Some items were scored as binary (0 = inability to perform, 1 = ability to perform), whilst others were scored as 0 = inability, 1 = adaptive and 2 = normal. Participants were allowed a rest period between items if needed. The POMA-G has a total of 12 marks: a score of < 9 indicates poor gait ability, 9–10 indicates good gait ability and 11–12 indicates excellent gait ability.^27^

### Procedure

#### Progressive resistance exercise group

A 12-week programme of progressive resistance excercise (PRE) was adopted from the American College of Sports Medicine guidelines for PLHIV.^28^ The exercise intervention was administered by senior physiotherapists specialised in exercise and sports sciences. The exercise session included light stretching of the quadriceps, gluteal muscles, hamstrings, tibialis anterior and calf muscles for 5 min to warm up. The proper training included strengthening of each of the following groups of muscles: quadriceps, gluteal, hamstrings, tibialis anterior and calf muscles. The participants performed strengthening exercises using the quadriceps bench (Enraf Nonius, Holland) with initial intensity set at 55% of one repetition maximum (1RM). The resistance level for each machine was gradually increased to 65% of 1RM to maintain the PRE effect. At the initial stage, each group of muscles performed two sets of 10 repetitions, with a resting period of 3 s – 5 s in between repetitions and a 5-min resting period in between the sets during the first 2 weeks. The set of 10 repetitions was increased to three for each muscle group during the remaining 10 weeks (weeks 3–12). The session ended with walking for 5 min to cool down. The exercise was conducted three times per week for three months. Vital signs were carefully monitored throughout the duration of the exercises. Borg rating of perceived exertion (RPE) is an outcome measure scale used to monitor progress and mode of exercise in individuals undergoing rehabilitation. The exertional rate was assessed using the Borg scale during the exercise training and immediately after the training period.^29^ Study outcomes were measured at baseline, 6 weeks and 12 weeks. All subjects continued on their ART drugs and to receive routine healthcare including ART drugs and medical consultation throughout the study.

#### Control group

The participants in this group were asked not to undertake any training or research involving exercise training for the period of the study. However, they also continued receiving routine healthcare including ART drugs and medical consultations.

### Statistical analysis

Statistical analyses were performed using IBM Statistical Package for Social Sciences (SPSS) (version 23.0, SPSS Inc., Chicago IL, United States), with normality being assessed using the Shapiro-Wilk Test and graphical methods. Normality of data distribution were not achieved; thus, the analyses were conducted using non-parametric statistics with the Mann-Whitney *U* test (for between groups) and the Friedman test (for baseline, 6-week and 12-week differences). The basic statistics were represented with median and interquartile ranges (IQR). An alpha level of < 0.05 was used to indicate significance.

## Results

The age range of the participants was 20–55 years. The mean (± standard deviation [± s.d.]) age of the experimental and CG were 35.98 (± 8.53) and 36.13 (± 8.10) years, respectively. The socio-demographic and basic health status characteristics of the participants were similar in all baseline measures between the groups (Table 1a and 1b). Seven (6.9%) of the 51 participants randomised to the PRE group dropped out, whilst four (3.9%) of the 51 randomised to the CG dropped out (Figure 1). Exercise subjects who did not drop out had excellent adherence attending 95% of scheduled exercise sessions.

**TABLE 1a:** Socio-demographic status of the respondents.

Characteristics	All participants(*N* = 91)		PRE (*N* = 44)		CG (*N* = 47)		*t*	*p*
	Mean	s.d.	Mean	s.d.	Mean	s.d.		
Age (years)	36.05	8.03	35.98	8.53	36.13	8.10	1.114	0.331
Weight (kg)	68.42	16.39	70.96	15.81	70.20	16.21	2.437	0.091
Height (m)	1.67	0.11	1.67	0.11	1.67	0.12	0.042	0.959

PRE, progressive resisted exercise; CG, control group; s.d., standard deviation.

**TABLE 1b:** Socio-demographic status of the respondents.

All participants (*N* = 91)	PRE (*N* = 44)		CG (*N* = 47)		*U*	*p*
	*N*	%	*N*	%		
**Gender**						
Male	20	45.5	18	38.3	0.51	0.773
Female	24	54.5	29	61.7	^-^	^-^
**How infected**						
Having sex with men	16	36.4	25	53.2	0.701	0.522
Having sex with women	19	43.2	16	34.0	^-^	^-^
Injecting drugs	7	15.9	1	2.1	^-^	^-^
Exposure to blood products	2	4.5	5	10.6	^-^	^-^
**Level of education**						
No formal education	4	9.1	25	53.2	1.603	0.449
Primary school	4	9.1	16	34.0	^-^	^-^
Secondary school	24	54.5	1	2.1	^-^	^-^
Tertiary education	12	27.3	5	10.6	^-^	^-^
**Health status**						
Very poor	16	36.4	11	23.4	1.012	0.603
Poor	24	54.5	33	70.2	^-^	^-^
Neither poor nor good	4	9.1	3	6.4	^-^	^-^
Good	0	0.0	0	0.0	^-^	^-^
**Marital status**						
Single	12	27.3	8	17.1	1.856	0.10
Married	14	31.8	19	40.4	^-^	^-^
Widow or widower	8	18.2	11	23.4	^-^	^-^
Divorced/separated	10	22.7	9	19.1	^-^	^-^
**Duration since AIDS diagnosis**						
≤ 3 years ago.	6	13.6	7	14.9	^-^	0.64
4–6 years ago	10	22.7	13	27.7	^-^	^-^
≥ 7 years ago	28	63.6	27	57.4	^-^	^-^
**Duration on ARVs**						
≤ 3 years ago.	10	22.7	8	17.1	0.875	0.96
4–6 years ago	27	61.4	30	63.8	^-^	^-^
≥ 7 years ago	7	15.9	9	19.1	^-^	^-^
**ARV regimen combination started with**						
Not on D4T	24	54.5	28	59.6	1.702	0.87
On D4T	20	45.5	19	40.4	^-^	^-^
**Current ARV regimens’ combination**						
Non-4T including	37	84.1	39	83.0	1.547	0.77
D4T including	7	15.9	8	17.0	^-^	^-^
**ARV regimen changes since started**						
No changes	10	22.7	13	27.7	0.7743	0.12
Once or more changes	34	7.3	34	72.3	^-^	^-^
**The onset of DSPN symptoms and signs**						
Before starting on ARVs	5	11.4	9	19.1	0.321	0.082
After starting on ARVs	39	88.6	38	80.9	^-^	^-^
**After how long on ARVs when DSPN started**						
Within the first 12 months	20	45.5	19	40.4	0.567	0.69
After the first 12 months	24	54.5	28	59.6	^-^	^-^

Average CD4 (cells/mm2): PRE Median = 11, PRE IQR = 1.0; CG Median = 10, CG IQR = 3.0; *U* = 11.022; *p* = 0.00.

U, Mann Whitney Test; *t, T*-test; ARV, antiretroviral; D4T, stavudine; DSPN, Distal symmetrical polyneuropathy; PRE, progressive resistance exercise; CG, control group ; IQR, interquartile range.

Significant differences were observed for balance measures across the PRE group (*p* < 0.05; Table 2). Post-hoc analysis of Wilcoxon signed-rank test showed significant improvement from the baseline to 6 weeks of intervention (effect size *r* = 0.88, *p* = 0.001), from baseline to 12 weeks post-intervention (effect size 0.88, *p* = 0.001) and from 6 weeks to 12 weeks post-intervention (effect size *r* = 0.86, *p* = 0.001). In contrast, the CG experienced no significant improvement (*p* > 0.05).

**TABLE 2:** Differences within groups in balance and gait scores across baseline, 6 weeks and 12 weeks.

Variables	Study groups	*N*	Baseline		6 weeks		12 weeks		*X* ^2^	*p*
			Median	IQR	Median	IQR	Median	IQR		
Balance	PRE	44	10.0	8.00–12.00	12.0	9.20–13.30	15.00	13.6–18.30	87.034	< 0.001*
	CG	47	10.0	7.10–11.80	9.00	7.00–11.00	9.00	7.08–10.60	11.333	0.513
Walking gait	PRE	44	7.00	5.60–9.10	8.00	6.20–10.50	11.00	10.0–13.70	67.304	< 0.001*
	CG	47	8.00	6.30–10.70	8.00	6.30–11.00	8.00	6.50–10.90	8.797	0.12

PRE, progressive resistance exercise; CG, control group; IQR, interquartile range; *X*^2^, Chi square of Wilcoxon signed-rank test.

* , Significant at *p* < 0.05.

Also, significant improvements for gait ability measures were observed across the PRE group (Table 2). Post-hoc analysis of Wilcoxon signed-rank test indicated significant improvement from baseline to 6 weeks (effect size *r* = 0.56, *p* = 0.001), from baseline to 12 weeks (effect size = 0.87, *p* = 0.001) and from 6 weeks to 12 weeks (effect size *r* = 0.79, *p* = 0.001). No positive changes in gait measures were observed over time in the CG (*p* < 0.05).

At 6 and 12 weeks of the intervention, statistically significant differences were observed between the experimental and the CG in balance measures with effect size *r* = 0.71, *p* = 0.001 and effect size *r* = 0.85, *p* = 0.001 respectively; and for gait measures with effect size *r* = 0.29, *p* = 0.006 and effect size *r* = 0.63, *p* = 0.001 (Table 3).

**TABLE 3:** Between-groups comparison of the outcomes at baseline, 6 weeks and 12 weeks.

Variable	Time period	PRE (*n* = 44)		CG (*n* = 47)		*U*	*p*
		Median	IQR	Median	IQR		
Balance	Baseline	10.0	8.20–12.00	10.0	7.80–12.10	1016.000	0.677
	6 weeks	12.0	9.20–14.00	9.00	8.00–11.00	193.500	< 0.001*
	12 weeks	15.0	12.10–17.00	9.00	7.80–11.60	19.000	< 0.001*
Walking (gait)	Baseline	7.00	5.30–9.00	8.00	6.20–9.00	696.000	0.578
	6 weeks	9.00	7.40–11.30	8.00	6.40–9.00	965.000	0.006*
	12 weeks	10.0	7.30–13.00	8.00	7.00–9.60	290.000	< 0.001*

U, Mann–Whitney Test; IQR, interquartile range; PRE, progressive resistance exercise; CG, control group.

* , Significant at *p* < 0.05.

## Discussion

This study was conducted to evaluate the effect of strength training on balance impairment and gait disturbance in individuals affected with HIV-related neuropathy. The exercise intervention improved balance impairment and gait disturbance in people living with HIV-related DSPN on ART who exercised for three months. The exercise group demonstrated significant improvement from six weeks for both balance impairment and walking ability.

The findings of this study prove the beneficial effect of therapeutic exercise on neuromuscular pathology causing impairment and disability, and affecting QOL. There is limited existing information on the impact of exercise on balance impairment and gait disturbance amongst HIV-positive individuals who develop neuropathy. With regard to gait disturbance, the result of the current study corroborates the work of Allet et al.^30^ who used 12 weeks of exercise to improve the gait performance of persons with diabetes. Significant favourable changes were observed in the gait performance of participants following a supervised strength exercise programme. In contrast, Kruse et al.^31^ found no positive changes in balance or strength in patients with diabetic neuropathy following supervised PRE for 60 min, twice a week for 12 weeks. They concluded that it could be because the treatment administered was not intense enough to produce a positive effect on balance and muscular strength. However, Graham et al.^32^ observed significant improvement in balance impairment in patients with non-HIV associated neuropathy after 12 weeks of unsupervised, community-based strengthening exercise, agreeing with the positive change noted in balance and walking ability following PRE in the current study. Richardson, Sandman and Velas^33^ subjected patients with diabetic PN to PRE for three weeks every day for 35 min – 50 min per session using 60% – 70% of 1RM. Significant improvement was observed in clinical measures of postural performance (*p* < 0.05), which corroborates the finding of this study.

It has been stated that the strengthening of groups of muscles around the knee joint is related to positive changes in stride length and cadence during walking and can influence balance performance, in general. It is an intervention that can also improve walking ability.^34^ Judge, Underwood and Gennosa^35^ found that in older individuals with PN (mean age 82.1 years), 12 weeks of resistance exercises three times per week produced a significant modulating effect on strength, balance parameters and gait velocity when compared with a CG that performed only flexibility exercises (*p* < 0.05), tallying with results from the current study. These results accord with the postulated literature that states that resistance exercises can attenuate motor deficits caused by PN, thus improving inter- and intra-muscular coordination as well as neural control, and leading to improvement in balance impairment and gait disturbance.^36^

## Conclusion

This research study provided insights into the use of moderate-intensity strength exercises and the use of progressive resistance. The clinical implication is that PRE may be of use to physiotherapists when managing individuals with HIV-related DSPN for whom exercises are not contraindicated. This study supports the safety of moderate-intensity PRE and the improvement of impaired balance and walking disturbance in PLHIV-related DSPN. The use of PRE could minimise residual disabilities from HIV-related DSPN. This research study may serve as a starting point for further studies of larger sample size and longer duration.

### Limitations

Lack of diagnostic tools such as electromyogram (EMG) or nerve conducting study to confirm the presence of neuropathy was one of the study’s limitations, as the study was conducted in a poor resource environment. The authors included both participants with symptoms before starting antiretrovirals (ARVs) and whilst starting ARVs. However, the tools used in this study were validated and reliable for the diagnosis of HIV-related DSPN. Also, participants assigned to the intervention group could not be blinded to the type of interventions, and the generalisability of the results is limited because of the use of a non-probability sampling technique. Furthermore, information was not available on follow-up post-intervention to assess any long-term changes in the functional status.
